# Microvascular Coronary Artery Spasm Presents Distinctive Clinical Features With Endothelial Dysfunction as Nonobstructive Coronary Artery Disease

**DOI:** 10.1161/JAHA.112.002485

**Published:** 2012-10-25

**Authors:** Keisuke Ohba, Seigo Sugiyama, Hitoshi Sumida, Toshimitsu Nozaki, Junichi Matsubara, Yasushi Matsuzawa, Masaaki Konishi, Eiichi Akiyama, Hirofumi Kurokawa, Hirofumi Maeda, Koichi Sugamura, Yasuhiro Nagayoshi, Kenji Morihisa, Kenji Sakamoto, Kenichi Tsujita, Eiichiro Yamamoto, Megumi Yamamuro, Sunao Kojima, Koichi Kaikita, Shinji Tayama, Seiji Hokimoto, Kunihiko Matsui, Tomohiro Sakamoto, Hisao Ogawa

**Affiliations:** Department of Cardiovascular Medicine, Faculty of Life Sciences, Graduate School of Medical Sciences, Kumamoto University, Kumamoto, Japan (K.O., S.S.,H.S., T.N., J.M., Y.M.,M.K., E.A.,H.K., H.M.,K. Sugamura, Y.N., K.Morihisa, K. Sakamoto, K.T., E.Y.,M.Y., S.K., K.K., S.T., S.H., T.S., H.O.); Department of General Medicine, Yamaguchi University Hospital, Ube, Japan (K. Matsui)

**Keywords:** angina, follow-up studies, microcirculation, vasospasm, women

## Abstract

**Background:**

Angina without significant stenosis, or nonobstructive coronary artery disease, attracts clinical attention. Microvascular coronary artery spasm (microvascular CAS) can cause nonobstructive coronary artery disease. We investigated the clinical features of microvascular CAS and the therapeutic efficacy of calcium channel blockers.

**Methods and Results:**

Three hundred seventy consecutive, stable patients with suspected angina presenting nonobstructive coronary arteries (<50% diameter) in coronary angiography were investigated with the intracoronary acetylcholine provocation test, with simultaneous measurements of transcardiac lactate production and of changes in the quantitative coronary blood flow. We diagnosed microvascular CAS according to lactate production and a decrease in coronary blood flow without epicardial vasospasm during the acetylcholine provocation test. We prospectively followed up the patients with calcium channel blockers for microvascular coronary artery disease. We identified 50 patients with microvascular CAS who demonstrated significant impairment of the endothelium-dependent vascular response, which was assessed by coronary blood flow during the acetylcholine provocation test. Administration of isosorbide dinitrate normalized the abnormal coronary flow pattern in the patients with microvascular CAS. Multivariate logistic regression analysis indicated that female sex, a lower body mass index, minor–borderline ischemic electrocardiogram findings at rest, limited–baseline diastolic-to-systolic velocity ratio, and attenuated adenosine triphosphate–induced coronary flow reserve were independently correlated with the presence of microvascular CAS. Receiver-operating characteristics curve analysis revealed that the aforementioned 5-variable model showed good correlation with the presence of microvascular CAS (area under the curve: 0.820). No patients with microvascular CAS treated with calcium channel blockers developed cardiovascular events over 47.8±27.5 months.

**Conclusions:**

Microvascular CAS causes distinctive clinical features and endothelial dysfunction that are important to recognize as nonobstructive coronary artery disease so that optimal care with calcium channel blockers can be provided.

**Clinical Trial Registration:**

URL: www.umin.ac.jp/ctr. Unique identifier: UMIN000003839.

## Introduction

Coronary angiography (CAG) frequently reveals nonobstructive coronary arteries in patients with suspected angina.^1,2^ Nonobstructive coronary artery disease (CAD), which accounts for 15% of non–ST-elevated cases of acute coronary syndrome,^3^ has attracted much clinical attention because it is a high-risk condition with an incidence of annual adverse cardiovascular events of >5%.^4,5^ Nonobstructive CAD should be diagnosed as angina with epicardial coronary artery spasm (epicardial CAS)^6^ or as microvascular CAD, such as microvascular coronary artery spasm (microvascular CAS),^7^ microvascular coronary dysfunction,^8,9^ and the other causes of myocardial ischemia.^10,11^ Angiographic assessment via the intracoronary acetylcholine-provocation test (ACh test) is the standard method for diagnosing epicardial CAS in nonobstructive CAD.^12^ However, the presence of microvascular CAS has not been well recognized in clinical practice, and the clinical characteristics and features remain to be determined because of the lack of an objective assessment method for detecting microvascular CAS–induced myocardial ischemia. Recently, Ong et al reported a high prevalence of microvascular CAS in white patients with stable angina pectoris with nonobstructive coronary arteries.^13^

Increased lactate production in the coronary circulation is a definitive sign of myocardial ischemia,^14^ and it is possible to compare plasma lactate levels in the aortic root and the coronary sinus to assess the occurrence of myocardial ischemia during CAG. In addition, we can continuously monitor and evaluate increases or decreases in the coronary blood supply by measuring the quantitative coronary blood flow (CBF) by using the Doppler technique with an intracoronary Doppler-tipped guidewire. The ACh test is an endothelium-dependent coronary reactivity test to assess endothelial function and CAS. In contrast, as a non–endothelium-dependent coronary reactivity test, adenosine triphosphate–induced coronary flow reserve (ATP-CFR) is also useful for diagnosing microvascular coronary dysfunction in nonobstructive CAD.^9^ Overall, a comprehensive clinical diagnostic procedure combined with the ACh test and ATP-CFR enables us to examine various types of nonobstructive CAD in current clinical cardiovascular practice.

We hypothesized that microvascular CAS could be accurately diagnosed as angina without obstructed coronary lesions, corresponding to myocardial ischemia without an angiographic spasm in the epicardial coronary arteries. We examined whether the diagnosis could be made on the basis of decreases in the CBF detected with the ACh test. We performed the ACh test in patients undergoing CAG and simultaneously measured lactate production and CBF changes to investigate the incidence, clinical characteristics, and factors associated with the presence of microvascular CAS in patients with angina-like chest symptoms and nonobstructive coronary arteries. Furthermore, we prospectively evaluated the therapeutic efficacy of calcium channel blockers (CCBs) in patients with microvascular CAS because CCB therapy is effective and clinically established for patients with epicardial CAS.^15^

## Methods

### Study Population

We recruited stable patients with suspected angina who were admitted to Kumamoto University Hospital. We excluded patients with possible heart failure (ejection fraction <50%) or previous diagnoses of CAD. From January 2002 to April 2011, 1105 consecutive and hemodynamically and symptomatically stable patients with suspected angina were registered. An initial CAG was performed to exclude obstructive CAD (≥50% diameter in major coronary arteries; n=718). Patients with angina-like chest symptoms and nonobstructive coronary arteries (<50% diameter) who completed the ACh test were enrolled in the present study (n=370).

### Study Protocol

**Figure 1.**
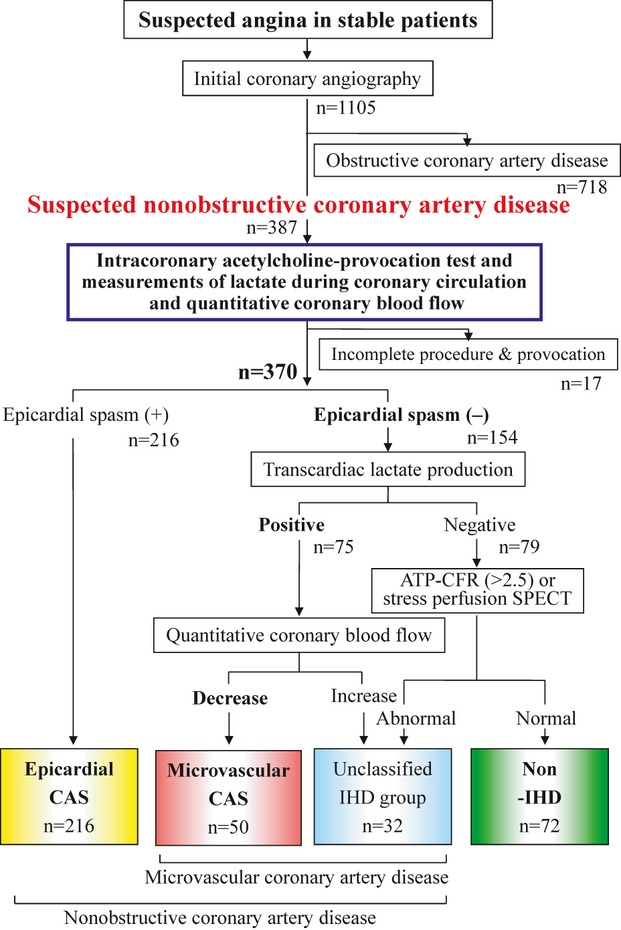
Diagnostic flowchart. The grouping of the patients in this study is shown. Microvascular coronary artery spasm (microvascular CAS) is diagnosed by the intracoronary acetylcholine-provocation test on the basis of the following criteria: positive for lactate production and a decrease in quantitative coronary blood flow without epicardial vasospasm, associated with the occurrence of chest symptoms and ischemic changes in the electrocardiogram. The numbers in the 4 boxes at the bottom of the diagram denote the number of patients in each group. The vertical lines connecting the boxes indicate the diagnostic processes. ATP-CFR indicates adenosine triphosphate–induced coronary flow reserve; epicardial CAS, epicardial coronary artery spasm; IHD, ischemic heart disease; and SPECT, single-photon emission computed tomography.

**Figure 2.**
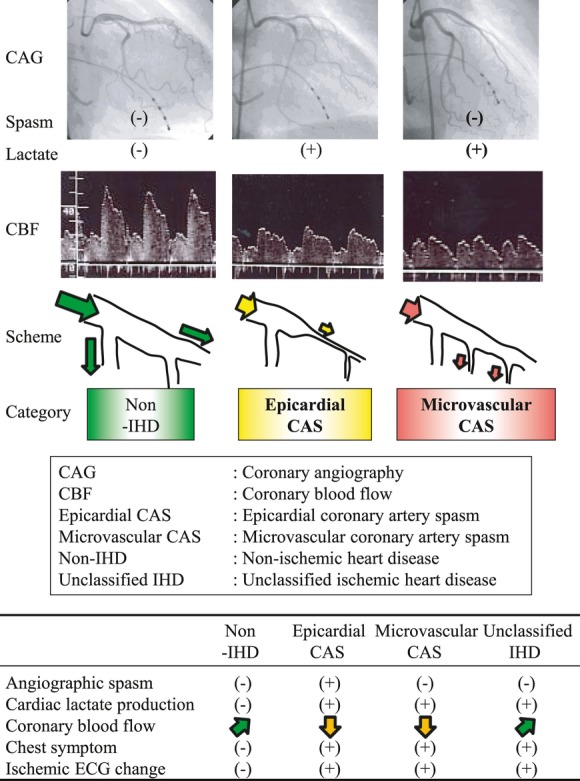
Scheme of categorization. This scheme shows the categorization of the non–ischemic heart disease (non-IHD), epicardial coronary artery spasm (epicardial CAS), and microvascular coronary artery spasm (microvascular CAS) in the intracoronary acetylcholine-provocation test. Microvascular CAS was defined as myocardial ischemia with the occurrence of chest pain, ischemic electrocardiogram changes, transcardiac lactate production, and a decrease in quantitative coronary blood flow (CBF) without epicardial coronary vasospasm using the acetylcholine-provocation test. CAG indicates coronary angiography; ECG, electrocardiogram.

**Figure 3.**
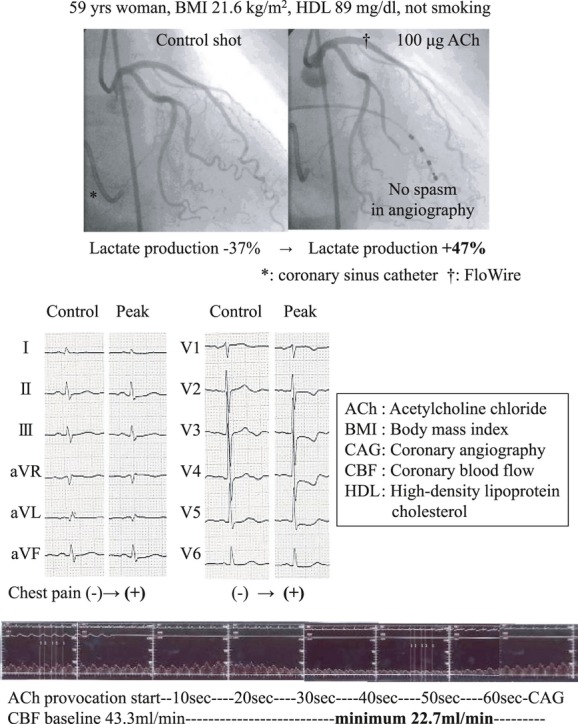
One case of microvascular coronary artery spasm. A representative case of microvascular coronary artery spasm is presented. Epicardial vasospasm is not induced by acetylcholine (ACh); however, the velocity of the coronary flow declines, thus, the quantitative coronary blood flow decreases. The transcardiac lactate production ratio then becomes positive during coronary circulation. Patients with microvascular coronary artery spasm showed decreases in the average peak velocity from baseline. BMI indicates body mass index; CAG, coronary angiography; CBF, coronary blood flow; and HDL, high-density lipoprotein-cholesterol.

We performed the ACh test with simultaneous measurements of lactate production and the quantitative CBF during the diagnostic CAG (Figure 1). Epicardial CAS was defined angiographically by severe vasospasm, which was visualized as lumen narrowing (>90%) in any of the major coronary arteries in response to ACh test, based on the Japanese Circulation Society's guideline for vasospastic angina.^12^ In the patients with nonepicardial CAS who were positive for lactate production, we diagnosed microvascular CAS on the basis of a decrease in CBF without epicardial vasospasm but with the occurrence of chest symptoms and ischemic changes in the electrocardiogram (ECG). In addition, the patients who were positive for lactate production but had no decrease in CBF were categorized as having unclassified ischemic heart disease (IHD). Patients who were negative for lactate production were further assessed by ATP-CFR and stress thallium-201 single-photon emission computed tomography. Patients with abnormal results on these tests ultimately were identified as having microvascular coronary dysfunction and also were categorized into the unclassified IHD group. Patients who had no abnormal results were defined as non-IHD patients (Figure 1). The categorization scheme is shown in Figure 2, and a representative case of microvascular CAS is depicted in Figure 3.

Written, informed consent was obtained from each patient before CAG and the comprehensive diagnostic analysis. This study was approved by the ethics committee of our institution and fully complied with the Declaration of Helsinki. A series of patients submitted to the diagnostic procedure were used as the case controls, and the follow-up results were registered in the Universal Hospital Medical Information Network Clinical Trials Registry (UMIN000003839; http://www.umin.ac.jp/ctr).

### Cardiac Catheterization

All vasoactive drugs, including CCBs, isosorbide dinitrate (ISDN), isosorbide mononitrate, nicorandil, and β-adrenergic–blocking agents, were discontinued ≥72 hours before the ACh test. We performed all examinations in the morning during fasting. After the baseline CAG, we inserted a 0.014-inch Doppler-tipped guidewire (FloWire; Volcano, Rancho Cordova, CA) into the proximal site of the left anterior descending coronary artery (LAD) (FloMap; Cardiometrics, Mountain View, CA).

### Intracoronary Acetylcholine-Provocation Test

Incremental doses (20, 50, and 100 μg) of ACh chloride were injected into the left coronary artery over a period of 30 seconds each, and CAG was performed 1 minute after the start of each provocation. The doses of ACh were administered at 5-minute intervals. Subsequently, 50 μg of ACh was injected into the right coronary artery without the FloWire, followed by CAG. The presence of an angiographic epicardial spasm was then evaluated according to the Japanese Circulation Society's guideline for vasospastic angina^12^ as described in the next section.

After the administration of intracoronary ISDN and CAG at an interval of 10 minutes, adenosine triphosphate (ATP; 150 μg/kg per minute) was administered via the central vein until maximal hyperemia was achieved for the calculation of ATP-CFR.^16^ ATP-CFR was calculated with the following formula: Hyperemia APV/Post-ISDN APV. APV indicates average peak velocity.

### Angiographical Identification of Epicardial CAS

For the identification of angiographic spasm of the epicardial coronary arteries, we applied the criteria described by the Japanese Circulation Society's guideline for vasospastic angina.^12^ In brief, a positive finding for coronary spasm on CAG in the ACh test is defined as “transient, total, or sub-total occlusion (>90% stenosis) of a coronary artery.” In addition, a definite diagnosis of vasospastic angina requires simultaneous signs or symptoms of myocardial ischemia (anginal pain and ischemic ECG change).

### Lactate Measurement During CAG

To assess myocardial ischemia on the basis of the detection of increased lactate production in the coronary circulation, paired blood samples were collected simultaneously from the aortic root (L_AR_) and the coronary sinus (L_CS_) by using a coronary sinus catheter at 3 time points: baseline, 1 minute after delivery of 100 μg of ACh via the left coronary artery, and after administration of ISDN. The lactate production ratio was calculated with the following formula: (L_CS_–L_AR_)/L_AR_×100 (%). Normally, the ratio is negative,^17^ and a positive value definitively indicates the occurrence of myocardial ischemia.

### Quantitative CBF Measurement

The APV and the diastolic-to-systolic velocity ratio (DSVR) of coronary flow were measured continuously by the intracoronary Doppler FloWire. The quantitative CBF of the proximal LAD was calculated as reported previously^18,19^: CBF = π (APV/2) (vessel diameter/2)^2^.

We calculated the maximum CBF at the highest APV during low-dose (20 μg) ACh provocation, which reflects the maximum endothelium-dependent coronary reactivity associated with the increase in blood flow. Using high-dose (50 or 100 μg) ACh provocation to detect the decline in CBF as a result of vasospasm or vasoconstriction with the lowest APV (increase or decrease) occurring within 40 to 60 seconds of the ACh provocation, we calculated the minimum CBF. This value enabled us to assess the decrease in blood flow by measuring the constriction response to ACh in the coronary arteries. The CBF was measured over a period of 40 to 60 seconds and was calculated from the APV, which was measured continuously, and the vessel diameter, which was considered to be same as that measured 1 minute after the ACh provocation.

### Quantitative CAG

The lumen diameter of the LAD at end diastole was measured with a computer-assisted coronary angiographic analysis system (CAAS 5.6; Pie Medical Imaging B.V., Maastricht, the Netherlands) by calibrating the measurement with a Judkins catheter, as previously reported.^20^

The LAD was divided into proximal, middle, and distal segments of equal length, and each luminal diameter was measured at the center of each segment. The coronary artery diameter was measured by 2 blinded investigators at 3 time points: baseline, after 1 minute of ACh provocation, and after 1 minute of ISDN administration.

### Electrocardiography

A digital 12-lead ECG was recorded by using an electrocardiogram recorder (FX-7542; Fukuda Denshi, Tokyo, Japan) when the patients were at rest with no chest symptoms. The results were read automatically with the Minnesota code^21^ and were interpreted for ischemic changes with minor–borderline findings by 2 cardiologists who were blinded to this study. The ECG was recorded before CAG in all patients. Minor–borderline ischemic ECG findings were defined as the presence of a borderline Q or QS wave (code I_3_), a significant or borderline ST-segment depression (code IV_1-3_), a deep or moderate T-wave inversion and a flat T wave (code V_1-3_), or a complete left bundle-branch block (code VII).

### Ultrasound Cardiography

The left ventricular ejection fraction, the left atrium diameter, the interventricle septal wall thickness, and the posterior wall thickness were measured by 2 sonographers on the basis of the recommendations for chamber quantification of the American Society of Echocardiology.^22^

In all patients, the ECGs and ultrasound cardiography were recorded at rest when no chest symptoms were present and before CAG.

### Biochemical Measurements

To measure the biochemical parameters, blood samples were obtained in the early morning after an overnight fast. B-type natriuretic peptide was measured with a commercially available assay (Abbott Japan Co, Matsudo, Japan). Renal function was determined from the estimated glomerular filtration rate, which was calculated by an equation recommended by the Japanese Society of Nephrology (mL/min per 1.73 m^2^).^23^

### Follow-Up Study

After the diagnosis of nonobstructive CAD, all patients with microvascular CAS were treated with CCBs and were instructed to use sublingual nitroglycerin (0.3 mg/tablet) for unexpected anginal attacks at home. The patients were followed up prospectively by cardiologists at outpatient clinics until May 2011 or until an endpoint occurred. Cardiovascular events were ascertained from a review of the medical records and were confirmed by direct contact with the patients and cardiologists.

An endpoint was defined as cardiovascular death, nonfatal myocardial infarction, nonfatal ischemic stroke, unstable angina pectoris, or hospitalization for congestive heart failure.

Cardiovascular death was defined as death due to myocardial infarction, congestive heart failure, stroke, or documented sudden cardiac death. A diagnosis of myocardial infarction was made via the detection of a rise or fall in the levels of cardiac biomarkers (plasma creatine kinase MB or cardiac troponin T) above the 99th percentile for the upper limit of normal variation, together with evidence of myocardial ischemia with at least one of the following: the presence of symptoms, changes in the ECG (new ST-T changes, left bundle-branch block, or pathological Q wave), imaging evidence of new loss of viable myocardium, or a new regional wall-motion abnormality.^24^ A diagnosis of ischemic stroke was made if the patient presented clinical and radiological evidence. A diagnosis of unstable angina pectoris was made on the basis of the presence of new or accelerating symptoms of myocardial ischemia accompanied by new ischemic ST-T-wave changes. A diagnosis of congestive heart failure was made if the patient was admitted with typical symptoms, typical signs, and objective evidence of a structural or functional abnormality of the heart at rest.

### Statistical Analysis

Continuous variables with a normal distribution are expressed as the mean±standard deviation. Continuous variables with a skewed distribution are expressed as the median (interquartile range). Statistical analysis was performed with the χ^2^ test for categorical variables.

Post hoc tests were performed to compare <3 groups, after 2-way analysis of variance, with the Fisher protected least-significant-difference procedure. Associations between the presence of microvascular CAS and the clinical characteristics were analyzed by univariate and multivariate logistic regression analyses with a backward-selection method (*P*<0.05 for stay) in patients with suspected nonobstructive CAD (n=370). For logistic regression analyses, the patients with epicardial CAS, the patients with unclassified IHD, and the non-IHD patients were included in the non–microvascular CAS group. The model calibration was tested with the Hosmer-Lemeshow test for the presence of microvascular CAS. Furthermore, we used the bootstrapping method to perform the receiver-operating characteristics curve and the area under the curve analyses for the presence of microvascular CAS with the selected variables.^25^ The area under the curve values were compared as previously reported.^26^ We defined the optimal thresholds of a 5-variable model index for the presence of microvascular CAS by maximizing the sum of the sensitivity and the specificity.^27^

Statistical significance was defined as *P*<0.05; all tests were 2 tailed. The statistical analyses were performed in SPSS version 17.0J for Windows (SPSS Inc, Chicago, IL) and STATA version 10 (Stata Corp, College Station, TX).

## Results

### Clinical Diagnosis of Microvascular CAS in Patients With Suspected Nonobstructive CAD

**Figure 4.**
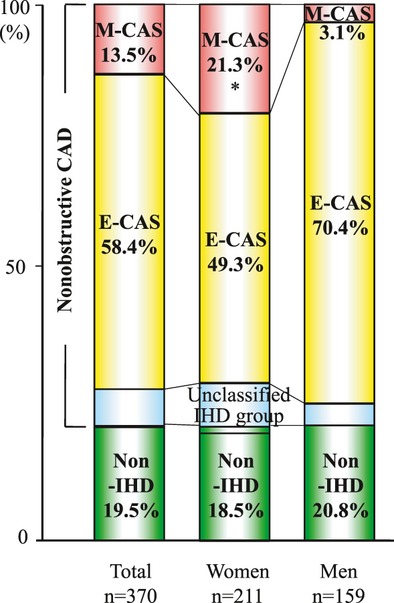
Percentage of patients with microvascular coronary artery spasm (M-CAS), epicardial coronary artery spasm (E-CAS), and non–ischemic heart disease (non-IHD) in patients with suspected nonobstructive coronary artery disease (nonobstructive CAD). M-CAS was significantly more frequent in women than in men (45 women [21.3%] vs 5 men [3.1%], *P<0.0001).

We identified patients with microvascular CAS on the basis of the current diagnostic procedures with simultaneous measurement of lactate production and CBF during the ACh test (Figure 1). In all 370 stable patients (211 women and 159 men) with angina-like chest symptoms and nonobstructive coronary arteries (suspected nonobstructive CAD), microvascular CAS was diagnosed in 50 cases by using the ACh test, and the patients exhibited normal CAG. Microvascular CAS was significantly more frequent in women than in men (45 women [21.3%] versus 5 men [3.1%]; *P*<0.0001, Figure 4). Seventy-two patients ultimately were diagnosed as non-IHD patients after the comprehensive diagnostic procedure with the ACh test and ATP-CFR.

### Baseline Clinical Characteristics

Of all the patients, those with microvascular CAS were more likely to be female, to have a lower body mass index, to be nonsmokers, to have a higher level of high-density lipoprotein cholesterol, and to exhibit minor–borderline ischemic ECG findings at rest. In all groups, there were more frequent angina-like chest symptoms at rest than during exertion, and we could not find any significant difference in the type of symptoms (Table 1). The chest symptoms at rest occurred more often at night and early in the morning than in the daytime and evening. The symptoms during exertion were more frequently observed in the morning and daytime than in the evening and night. We further classified the duration of the angina-like symptoms in all groups as short (<5 minutes), intermediate (5 to 10 minutes), and long (≥10 minutes) (Table 1), but there was no statistically significant difference. With regard to the effects of nitroglycerin, more than half of the patients did not have any experience of nitroglycerin use during the angina-like attacks before the ACh test; thus, we could not suitably analyze the clinical efficacy of nitroglycerin. Therefore, a differential diagnosis based on the medical history was difficult. The diagnostic CAG with the ACh test was clinically required for all patients to distinguish the angina-like symptoms from noncardiac origins. Most patients with microvascular CAS had shown negative findings on noninvasive stress tests.

**Table 1.** Baseline Clinical Characteristics*

Group	All (n=370)	Non-IHD (n=72)	E-CAS (n=216)	M-CAS (n=50)	U-IHD (n=32)
Age, y, mean±SD	63.0±11.2	62.6±11.8	62.9±11.1	62.7±10.6	64.7±10.8
Female, n (%)	211 (57.0)	39 (54.2)	104 (48.1)	45 (90.0)†‡	23 (71.9)
Body mass index, kg/m^2^	23.7±3.6	23.8±4.0	23.8±3.4	22.3±2.9†‡	24.5±4.2
Coronary risk factors					
Hypertension, n (%)	197 (53.2)	45 (62.5)	100 (46.3)†	31 (62.0)	21 (65.6)
Diabetes mellitus, n (%)	73 (19.7)	15 (20.8)	44 (20.4)	8 (16.0)	6 (18.8)
Dyslipidemia, n (%)	193 (52.2)	37 (51.4)	115 (53.2)	28 (56.0)	13 (40.6)
Smoking, n (%)	107 (28.9)	28 (38.9)	66 (30.6)	8 (16.0)†‡	5 (15.6)†
Familial history, n (%)	58(15.7)	8(11.1)	37(17.1)	11(22.0)	2(6.3)
Main angina situation					
Rest, n (%)	253 (68.4)	48 (66.7)	151 (69.9)	35 (70.0)	19 (59.4)
Exertion, n (%)	117 (31.6)	24 (33.3)	65 (30.1)	15 (30.0)	13 (40.6)
Anginal duration, min, n (%)					
<5	142 (38.4)	36 (50.0)	79 (36.6)	19 (38.0)	8 (25.0)
≥5 and <10	142 (38.4)	25 (34.7)	84 (38.9)	21 (42.0)	12 (37.5)
≥10	86 (23.2)	11 (15.3)	53 (24.5)	10 (20.0)	12 (37.5)
ECG findings, n (%)	141(38.1)	14 (19.4)	80 (37.0)†	33 (66.0)†‡	14 (43.8)†
Rhythm sinus/AF, n (%)	365/5 (1.4)	71/1 (1.4)	213/3 (1.4)	49/1 (2.0)	32/0 (0.0)
Biomarkers					
FPG, mg/dL	91 (83–98)	89 (83–100)	91 (84–98)	90 (82–105)	90 (82–94)
Hemoglobin A1c, %	5.8 (5.2–6.2)	5.8 (5.6–6.3)	5.8 (5.6–6.2)	6.0 (5.7–6.2)	5.7 (5.5–6.0)
Total cholesterol, mg/dL	194.4±35.3	185.3±40.9	195.3±33.6	203.5±32.5	195.8±32.4
LDL cholesterol, mg/dL	117.9±31.6	113.7±34.7	119.0±30.2	120.0±32.2	117.1±32.4
HDL cholesterol, mg/dL	56 (46–70)	53 (45–61)	55 (45–69)	63 (51–79)†‡	65(52–78)†
Triglyceride, mg/dL	111 (77–155)	110 (81–133)	112 (76–164)	96 (76–155)	97 (68–139)
eGFR, mL/min per 1.73 m^2^	74.4±17.4	71.8±18.2	75.2±17.4	72.6±13.9	78.2±19.3
BNP, pg/mL	20 (11–36)	22 (10–44)	20 (11–34)	20 (12–52)	18 (13–37)
hsCRP, mg/L	0.5 (0.3–1.2)	0.5 (0.2–1.1)	0.7 (0.3–1.4)	0.5 (0.2–0.9)	0.4 (0.2–1.1)
FRS, 10 years, %	8.0 (4.0–14)	8.0 (4.0–15)	8.0 (5.0–15)	6.0 (3.0–11)	6.0 (3.0–13)
Ultrasound cardiography					
Ejection fraction, %	66.0±6.5	64.8±6.9	65.8±6.5	67.0±5.3	68.4±7.5
Left atrium diameter, mm	35.5±5.4	35.5±5.6	35.6±5.3	34.4±5.6	36.2±5.5
Interventricle septal wall, mm	9.5±1.7	9.3±1.7	9.8±1.8	9.1±1.2	8.6±1.7
Posterior wall, mm	9.4±1.7	9.1±1.6	9.6±1.9	8.9±1.3	9.6±0.9
Noninvasive stress test for IHD					
Positive in TMT, n (%)	55/299 (14.9)	9/63 (12.5)	30/169 (13.9)	9/41 (18.0)	7/26 (21.9)
Positive in HVT, n (%)	12/253 (3.2)	0/54 (0.0)	12/142 (5.6)†	0/38 (0.0)‡	0/19 (0.0)
Positive in SPECT, n (%)	47/210 (12.7)	0/42 (0.0)	32/121 (14.8)	8/32 (16.0)	7/15 (21.9)
No or minor lesion in CAG					
Normal, n (%)	227 (61.4)	42 (58.3)	121 (56.0)	44 (88.0)†‡	19 (59.4)
Slight stenosis, n (%)	103 (27.8)	18 (25.0)	71 (32.9)	5 (10.0)‡	9 (28.1)
Moderate stenosis, n (%)	40 (10.8)	12 (16.7)	24 (11.1)	1 (2.0)	4 (12.5)
Coronary flow parameters (baseline)					
Diameter of LAD, mm	3.0±0.6	3.2±0.5	2.9±0.6†	3.2±0.4‡	3.0±0.5
APV, cm/s	21.7±7.0	19.4±5.5	22.1±7.7†	22.9±6.1†	22.7±6.0
CBF, mL/min	42.9±19.6	45.8±17.1	38.6±19.7†	46.3±19.8‡	50.7±20.8
DSVR	1.8±0.4	1.9±0.5	1.7±0.4	1.6±0.2†‡	2.0±0.6
Epicardial spasm during the Ach test					
LAD, n (%)	115/370 (31.1)	0 (0%)	115/216 (53.2)†	0 (0%)‡	0 (0%)
CX, n (%)	59/370 (15.9)	0 (0%)	59/216 (27.3)†	0 (0%)‡	0 (0%)
RCA, n (%)	108/336 (29.2)	0 (0%)	108/182 (50.0)†	0 (0%)‡	0 (0%)
Multivessel spasm, n (%)	87/336 (23.5)	0 (0%)	87/182 (40.3)†	0 (0%)‡	0 (0%)
ST-segment changes in the Ach test					
ST elevation, n (%)	81 (21.9)	…	72 (33.3)	6 (12.0)‡	3 (9.4)
ST depression, n (%)	217 (58.6)	…	144 (66.7)	44 (88.0)‡	29 (90.6)
ATP-CFR	3.1 (2.6–3.8)	3.3 (3.0–4.0)	3.1 (2.5–3.9)†	2.9 (2.5–3.2)†	2.8 (2.1–3.3)†

Each group indicates all patients (All), non–ischemic heart disease (non-IHD), epicardial coronary artery spasm (E-CAS), microvascular coronary artery spasm (M-CAS), and the unclassified-ischemic heart disease (U-IHD) group.

ACh indicates acetylcholine; AF, atrial fibrillation or atrial flutter; APV, average peak velocity; ATP-CFR, adenosine triphosphate–induced coronary flow reserve; BNP, B-type natriuretic peptide; CAG, coronary angiography after administration of isosorbide dinitrate; CBF, quantitative coronary blood flow; CX, circumflex artery; DSVR, diastolic-to-systolic velocity ratio; ECG findings, minor–borderline ischemic electrocardiogram findings at rest; eGFR, estimated glomerular filtration rate; FPG, fasting plasma glucose; FRS, Framingham Risk Score; HDL, high-density lipoprotein; hsCRP, high-sensitivity C-reactive protein; HVT, hyperventilation test; LAD, left anterior descending coronary artery; LDL, low-density lipoprotein; moderate stenosis, ≥25% and <50% diameter; multivessel spasm, angiographic spasm occurring in >1 vessel during the ACh test; RCA, right coronary artery; slight stenosis, <25% diameter; SPECT, single-photon emission computed tomography; and TMT, treadmill test.

* The data shown are the mean±standard deviation of the number of patients (percentage), or the median value (interquartile range).

† *P*<0.05 compared with the non-IHD group.

‡ *P*<0.05, M-CAS vs E-CAS.

Because endothelial dysfunction also can be an early sign of atherosclerosis, it is important to distinguish between normal and slightly or moderately abnormal angiograms. The CAG detected normal coronary arteries, slight stenosis (diameter <25%), and moderate stenosis (50% >diameter ≥25%) in all patients. The patients with microvascular CAS exhibited significantly fewer obstructive lesions (Table 1) than the other groups. Among the coronary flow parameters in the CAG, the patients with microvascular CAS demonstrated a faster APV, limited DSVR at the baseline condition, and attenuated ATP-CFR compared with the non-IHD patients (Table 1).

### Coronary Artery Response to Intracoronary ACh Provocation

In 34 epicardial CAS cases, coronary spasm required unscheduled treatment with intracoronary administration of ISDN (5 cases with 20 μg, 10 cases with 50 μg, and 19 cases with 100 μg) at the time of the ACh test for the left coronary artery; the ACh test could not be performed for the right coronary artery. During the ACh test, major adverse complications, such as coronary artery dissection, myocardial infarction, or cardiogenic shock, were not observed.

**Figure 5.**
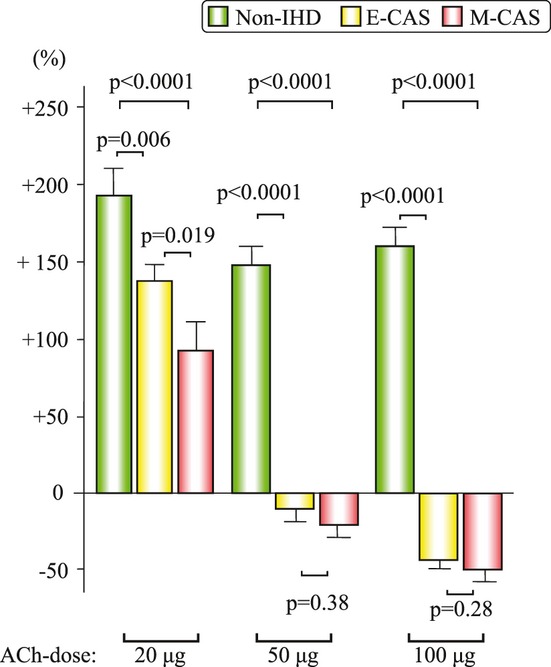
Changes in the quantitative coronary blood flow of the left anterior descending coronary artery in response to increasing doses of acetylcholine (ACh). The green bar shows non–ischemic heart disease (non-IHD), the yellow bar shows epicardial coronary artery spasm (E-CAS), and the red bar shows microvascular coronary artery spasm (M-CAS). The data shown are the mean±standard error of the mean of the percent change from each baseline.

**Figure 6.**
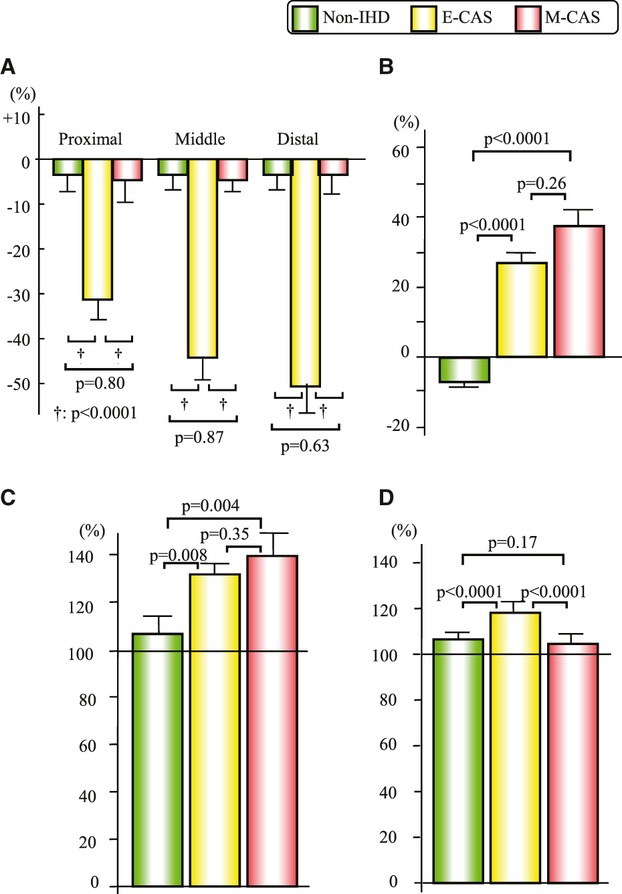
Changes in coronary artery parameters and the transcardiac lactate production ratio. A, Changes in the diameter of the left anterior descending coronary artery (LAD) in response to high-dose acetylcholine (ACh; 100 μg) in each segment. B, Transcardiac lactate production ratio in response to high-dose ACh. C, Changes in the diastolic-to-systolic velocity ratio in response to intracoronary administration of isosorbide dinitrate (ISDN). D, Changes in the diameter of the LAD in response to ISDN. The green bar shows non–ischemic heart disease (non-IHD), the yellow bar shows epicardial coronary artery spasm (E-CAS), and the red bar shows microvascular coronary artery spasm (M-CAS). The data shown are the mean ± standard error of the mean of the percent change from each baseline (A, C, and D).

In the low-dose intracoronary ACh provocation (20 μg), the increase in CBF was significantly smaller in the patients with microvascular CAS (+91.2±18.4%; *P*<0.0001) or epicardial CAS (+142.4±10.3%; *P*=0.006) than in non-IHD patients (+195.0±16.3%, Figure 5), indicating the presence of endothelial dysfunction in the patients with microvascular CAS. The non-IHD patients exhibited significantly increased CBF (+160.6±15.8%; *P*<0.0001) in response to high-dose ACh (100 μg). However, the patients with microvascular CAS and epicardial CAS showed significant and comparable decreases from baseline values in CBF at the LAD compared with the non-IHD patients (–54.3±5.0% and –41.7±4.7%, *P*=0.28, versus +160.6±15.8%, *P*<0.0001, respectively, Figure 5). At the high-dose ACh provocation, the patients with epicardial CAS but not the patients with microvascular CAS exhibited significantly greater vasoconstriction at the proximal, middle, and distal sites of the LAD than that of the non-IHD patients (*P*<0.0001, at each segment, Figure 6A).

### Myocardial Ischemia Assessed by Lactate Production and ECG

During the ACh test, the lactate production ratio was positive in the patients with microvascular CAS and epicardial CAS, in contrast to the negative value in the non-IHD patients (+38.7±6.6% and +26.5±4.4%, *P*=0.26, versus –9.2±0.9%, *P*<0.0001, respectively, Figure 6B). For the ECGs recorded during the ACh test, transient ST depression was significantly more frequent in patients with microvascular CAS. However, transient ST elevation was significantly more frequent in the patients with epicardial CAS (*P*=0.0005; Table 1).

### Coronary Artery Response to Intracoronary Administration of ISDN

The ACh-induced positive lactate production in the patients with epicardial CAS and microvascular CAS became significantly negative after ISDN administration (epicardial CAS: –11.0±1.5% and microvascular CAS: –10.2±2.2%). The change in the DSVR in response to ISDN was significantly greater in the patients with microvascular CAS and epicardial CAS than in the non-IHD patients (138.7±8.5% and 130.4±4.9%, versus 107.8±4.7%; *P*=0.004 and *P*=0.008, respectively, Figure 6C). The vasodilatory response to ISDN in the LAD from the baseline was significantly greater in the patients with epicardial CAS (119.7±1.7%) than in the non-IHD patients (107.8±1.1%, *P*<0.0001) and the patients with microvascular CAS (104.0±2.1%; *P*<0.0001, Figure 6D).

### Logistic Regression Analyses for the Presence of Microvascular CAS in Patients With Suspected Nonobstructive CAD

Of the 370 stable patients who were experiencing angina-like chest pain with nonobstructive coronary arteries (suspected nonobstructive CAD), the univariate logistic regression analysis demonstrated that female sex, minor–borderline ischemic ECG findings at rest, and high-density lipoprotein cholesterol levels were positively correlated with the presence of microvascular CAS, and the body mass index, smoking, Framingham risk score,^28^ baseline DSVR, and ATP-CFR were negatively correlated with the presence of microvascular CAS. Multivariate logistic regression analysis using the backward-selection method showed that the female sex (odds ratio [OR] 7.164; 95% confidence interval [CI], 1.883–27.26; *P*=0.004), body mass index (OR 0.843; 95% CI, 0.742–0.957; *P*=0.008), minor–borderline ischemic ECG findings at rest (OR 3.363; 95% CI, 1.408–8.033; *P*=0.006), the baseline DSVR (OR 0.788; 95% CI, 0.690–0.900; *P*=0.0004), and ATP-CFR (OR 0.941; 95% CI, 0.890–0.994; *P*=0.029) were independently correlated with the presence of microvascular CAS (Table 2). The Hosmer-Lemeshow goodness-of-fit χ^2^ value was 12.6 (*P*=0.13).

**Table 2.** Logistic Regression Analyses for the Presence of Microvascular CAS in Patients With Suspected Nonobstructive CAD*

Variables	Univariate Regression			Multivariate Regression		
	OR	95% CI	*P*	OR	95% CI	*P*
Age, y	1.003	0.976–1.030	0.85		Not selected	
Sex (female)	8.349	3.230–21.58	<0.0001	7.164	1.883–27.26	0.004
Body mass index, kg/m^2^	0.911	0.833–0.995	0.039	0.843	0.742–0.957	0.008
Hypertension (yes)	1.514	0.821–2.790	0.18		Not selected	
Diabetes mellitus (yes)	0.747	0.335–1.669	0.48		Not selected	
Dyslipidemia (yes)	1.196	0.656–2.178	0.56		Not selected	
Smoking (yes)	0.425	0.193–0.939	0.034		Not selected	
Familial history (yes)	1.638	0.784–3.424	0.19		Not selected	
Angina at rest (yes)	0.790	0.352–1.773	0.79		Not selected	
ECG findings (yes)	3.810	2.031–7.150	<0.0001	3.363	1.408–8.033	0.006
HDL cholesterol, mg/dL	1.024	1.006–1.041	0.008		Not selected	
eGFR, mL/min per 1.73 m^2^	0.990	0.973–1.008	0.28		Not selected	
BNP, pg/mL	1.002	0.997–1.008	0.34		Not selected	
hsCRP, mg/L	1.008	0.658–1.544	0.97		Not selected	
Framingham risk score, %	0.946	0.901–0.993	0.025		Not selected	
Baseline DSVR (per 0.1)	0.839	0.764–0.922	0.0003	0.788	0.690–0.900	0.0004
ATP-CFR (per 0.1)	0.952	0.918–0.988	0.009	0.941	0.890–0.994	0.029

This table shows the univariate and multivariate logistic regression analyses.

* OR indicates odds ratio; CI, confidence interval; not selected, not selected by the backward-selection method at a significance level of 0.05. Other abbreviations as in Table 1. The Hosmer-Lemeshow goodness-of-fit χ^2^ value was 12.6 (*P*=0.13).

### Receiver-Operating Characteristics Curve Analysis for the Presence of Microvascular CAS in Patients With Suspected Nonobstructive CAD

**Figure 7.**
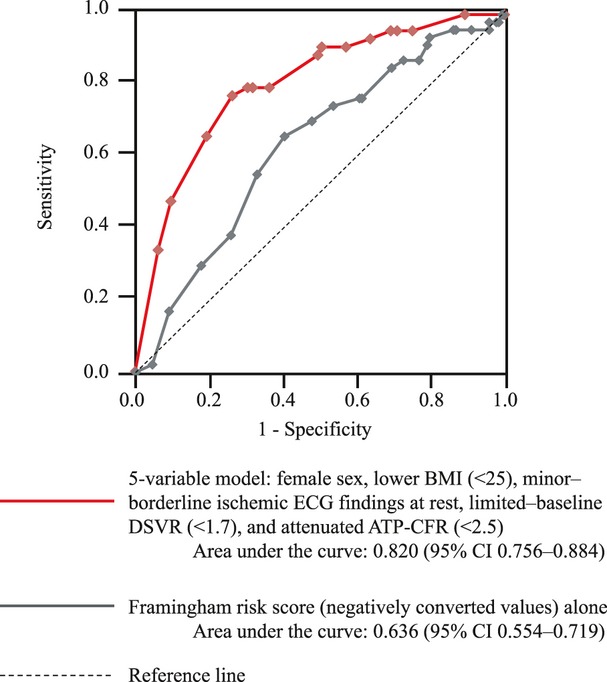
Receiver-operating characteristics curve analysis in the presence of microvascular coronary artery spasm (microvascular CAS) in patients with suspected nonobstructive coronary artery disease (CAD). The receiver-operating characteristic curve analysis was performed to assess the ability of the 5-variable model (female sex, lower body mass index [BMI <25 kg/m^2^], minor–borderline ischemic electrocardiogram [ECG] findings at rest, a limited–baseline diastolic-to-systolic velocity ratio [DSVR <1.7], and an attenuated adenosine triphosphate–induced coronary flow reserve [ATP-CFR <2.5]) and a Framingham risk score (negatively converted values) alone to predict the presence of microvascular CAS among the patients with suspected nonobstructive CAD. The area under the curve for the prediction of microvascular CAS was 0.820 (95% confidence interval [CI], 0.756–0.884; *P*<0.0001) for the 5-variable model and 0.636 (95% CI, 0.554–0.719; *P*=0.003) for a negative Framingham risk score, which were significantly different from each other (*P*=0.0004).

Receiver-operating characteristics curve analysis was used to assess the ability of the 5-variable model (sex, body mass index, minor–borderline ischemic ECG findings at rest, baseline DSVR, and ATP-CFR) and the Framingham risk score (negatively converted values) to identify the presence of microvascular CAS in patients with suspected nonobstructive CAD. The area under the curve for the prediction of microvascular CAS was 0.820 (95% CI, 0.756–0.884; *P*<0.0001) for the 5-variable model and 0.636 (95% CI, 0.554–0.719; *P*=0.003) for the Framingham risk score (negatively converted values), which were significantly different from each other (*P*=0.0004, Figure 7). With a 5-variable model index cutoff value of 9.8, the sensitivity and specificity for the presence of microvascular CAS were 75% and 80%, respectively.

### Follow-Up Study

All patients with microvascular CAS were treated with CCBs. The patients with microvascular CAS were followed up (n=49) for 47.8±27.5 months, until May 2011, with the exception of 1 patient who was lost to follow-up. The angina attacks disappeared completely in half of the patients with microvascular CAS after CCB therapy. Approximately 20% of the patients with microvascular CAS exhibited a few chest pain attacks within 1 year, and the remaining 30% of the patients exhibited several symptoms within 1 year. Forty percent of the angina attacks improved spontaneously, and 60% of attacks required sublingual nitroglycerin. No patients exhibited additional ischemic ECG changes or elevation in ischemic markers, including plasma creatine kinase MB or cardiac troponin T, during the follow-up period. There was no instance of cardiovascular death, nonfatal myocardial infarction, nonfatal ischemic stroke, unstable angina pectoris, or hospitalization for congestive heart failure.

## Discussion

The present study reveals that patients with microvascular CAS demonstrate distinctive clinical features related to coronary endothelial dysfunction during angina-like chest symptoms. We found that female sex, a lower body mass index, minor–borderline ischemic ECG findings at rest, limited–baseline DSVR, and attenuated ATP-CFR were independently associated with the presence of microvascular CAS. Furthermore, we found that CCBs were effective for treating patients with microvascular CAS.

In this study, all 50 patients with microvascular CAS presented angina accompanied with significant positive lactate production without epicardial stenosis or vasospasm associated with an ACh-induced decrease in CBF. Previously, Mohri et al identified 29 patients (9 with lactate production) and Ong et al identified 42 patients (no evidence of lactate production) as having microvascular CAS with angina-like chest pain and ischemic changes in the ECG without epicardial stenosis or vasospasm during the ACh test.^7,13^ However, the authors could not objectively demonstrate actual decreases in CBF without spastic changes in epicardial arteries, and they did not specifically investigate evidence of myocardial ischemia using metabolic markers. We quantitatively investigated transient decreases in CBF leading to myocardial ischemia by using intracoronary Doppler FloWire and assessed the occurrence of ischemia by measuring lactate production during an ACh test. Thus, we could objectively examine and diagnose an angina “microvascular CAS” on the basis of biochemical and hemodynamic evidence. Using the current comprehensive diagnostic procedure, we carefully and successfully diagnosed 50 patients with microvascular CAS, and this condition was confirmed to be an important clinical entity that accounted for 20% of the female patients having angina-like chest symptoms with nonobstructive coronary arteries.

Because conventional CAG could identify only epicardial CAS during the routine ACh test, it is evident that patients with microvascular CAS are underdiagnosed in clinical practice. It is reported that >15% of patients, especially women,^3^ with non–ST-elevation acute coronary syndrome present with nonobstructive coronary arteries. In addition, many cases of coronary artery vasospasm recently have been documented, primarily by use of CAG, throughout the world.^29^ Recently, a high prevalence of microvascular CAS has been demonstrated in white patients with stable angina pectoris, but an effective therapy was not determined.^13^ It has been reported that treatment with CCBs could improve ischemic symptoms and the prognoses of patients with epicardial CAS.^30^ In the present study, we demonstrated that patients with microvascular CAS exhibited a good prognosis after treatment with CCBs, thus indicating a potential therapeutic strategy for patients with microvascular CAS. It is important to recognize the presence of microvascular CAS in practice, and the various causes of angina should be examined in detail. Effective and optimal treatments for each individual patient will then be possible.

The modalities for diagnosing obstructive CAD have improved remarkably. However, they are insufficient to evaluate physiological and functional abnormalities in the coronary circulation, including the microvasculature. When coronary artery functions are evaluated for the diagnosis of IHD, the ACh test, ATP-CFR, magnetic resonance imaging, positron emission tomography, or stress perfusion scintigraphy is required.^31^ For the diagnosis of microvascular CAS, a FloWire is useful to directly and precisely assess the real-time and transient decrease in CBF. The baseline DSVR at rest has been proposed as a useful indicator of severe epicardial obstructive stenosis but not of nonobstructive CAD. The present study is the first to indicate that the limited–baseline DSVR is significantly correlated with abnormal microcirculation in nonobstructive coronary arteries.

The mechanisms underlying microvascular CAS have not been well characterized and remain inconclusive at the present time. We found that women were more frequently diagnosed with microvascular CAS, and therefore, the clinical features of this condition might represent a relatively specific vascular response in females. The clinical features of microvascular CAS, such as female sex, middle–old age, and a low body mass index, are reminiscent of postmenopausal phenomena, such as hip fracture, rather than vascular inflammation or atherosclerosis, which suggests a potential link with age-dependent estrogen deficiency in the pathogenesis of microvascular CAS. Unfortunately, estrogen levels were not examined in this study. We have reported recently that peripheral endothelial dysfunction is correlated with the presence of nonobstructive CAD in women.^32^ Patients with microvascular CAS exhibited significant impairment of the coronary endothelium-dependent vascular response assessed by CBF during the Ach test, indicating the dysregulation of the coronary microcirculatory system in microvascular CAS, in part via endothelial dysfunction. Additionally, ISDN improved the limited DSVR in patients with microvascular CAS, which suggests the possible involvement of nitric oxide deficiency or the impaired bioavailability of nitric oxide in coronary prearterioles (>200 μm), similar to that which occurs at epicardial arteries in patients with epicardial CAS.^33^ The patients with microvascular CAS showed attenuated ATP-CFR compared with non-IHD patients, but the ATP-CFR values of patients with microvascular CAS were almost within the normal range and displayed a broad overlap with those of non-IHD patients. Therefore, microvascular CAS cannot be diagnosed simply by ATP-CFR alone, and it could display a different pattern of pathogenesis from microvascular coronary dysfunction with impaired ATP-CFR.

CCB therapy is effective for treating epicardial CAS,^12^ and the prognosis of epicardial CAS treated with CCBs previously has been shown to be good. However, the efficacy of CCBs had not been established previously for microvascular CAS.^34^ Additionally, for microvascular coronary dysfunction assessed by ATP-CFR, as advocated by Merz and Pepine,^8^ the clinical outcome in patients with microvascular coronary dysfunction was improved by β-blockers or angiotensin-converting enzyme inhibitors but not by CCBs.^9^ In the present study we found, for the first time, that the prognosis for microvascular CAS patients is good after receipt of a definitive diagnosis, treatment with CCBs, and a cardiologist's close follow-up, similar to patients with obstructive CAD, which indicates that CCBs could be clinically useful for the treatment of microvascular CAS. CCBs could prevent an abnormal constriction response in the prearterioles with coronary endothelial dysfunction in microvascular CAS.

The present study has some limitations. This is a small, single-center study. This procedure allowed the diagnosis of microvascular CAS only for the LAD. However, microvascular CAS could occur in another artery or in all coronary arteries. Microvascular CAS should be evaluated in future studies to determine the external validity of the present prediction model in other patients. Because the follow-up study had no control group to compare with CCBs, the efficacy of the CCB treatment for patients with microvascular CAS should be further examined with groups who receive no treatment or another treatment, in different populations. We did not observe microvascular spasms directly, but we obtained a reasonable estimate of the occurrence of microvascular spasms based on a decrease in CBF. We also diagnosed microvascular spasms as angina on the basis of evidence for ischemia in the absence of spastic changes in CAG.

In conclusion, microvascular CAS in nonobstructive coronary arteries demonstrated distinctive clinical features and coronary endothelial dysfunction. With accurate diagnosis and optimal treatment with CCBs, the prognosis in patients with microvascular CAS is good. Thus, the comprehensive physiological diagnosis of nonobstructive CAD, including microvascular CAS, has progressed beyond anatomic evaluation by CAG to assess abnormalities via the coronary circulation, leading to improvements in patient care.
